# Correction: Golovko et al. Ambient Dose and Dose Rate Measurement in SNOLAB Underground Laboratory at Sudbury, Ontario, Canada. *Sensors* 2023, *23*, 1945

**DOI:** 10.3390/s24165274

**Published:** 2024-08-14

**Authors:** Victor V. Golovko, Oleg Kamaev, Jiansheng Sun, Chris J. Jillings, Pierre Gorel, Eric Vázquez-Jáuregui

**Affiliations:** 1 Canadian Nuclear Laboratories, 286 Plant Road, Chalk River, ON K0J 1J0, Canada; 2 SNOLAB, Lively, ON P3Y 1N2, Canada; 3 Instituto de Física, Universidad Nacional Autónoma de México, A. P. 20-364, Mexico City 01000, Mexico

We have found a discrepancy in the reported exposure time for passive sensors, specifically TLDs, in the original manuscript entitled “Ambient Dose and Dose Rate Measurement in SNOLAB Underground Laboratory at Sudbury, Ontario, Canada” [1]. This discrepancy only affects the dose rate values, not the measured ambient dose. We have made corrections to the affected rate values.

## Text Correction

In the first paragraph of Section 3, the sentence “Detectors were placed in Cube Hall at SNOLAB on Thursday, 27 December 2018 around 2 p.m. and were taken out on Tuesday, 22 January 2019 around noon”. has been updated to “Detectors were placed in Cube Hall at SNOLAB on Thursday, 27 November 2018 around 2 p.m. and were taken out on Tuesday, 22 January 2019 around noon”.

In the second paragraph of Section 3, the phrase “ambient dose measurements in Cube Hall was 25 days and 22 h, or 622 h” has been updated to “ambient dose measurements in Cube Hall was 55 days and 22 h, or 1342 h”. The next phrase in the same paragraph “the deployment time interval of the passive integrating detectors was 622 ± 2 h” has been updated to “the deployment time interval of the passive integrating detectors was 1342 ± 2 h”.

In the second paragraph of Section 4, the last sentence “In other words, the dose data from Table 1 for badge IDs from 4 to 24 and badge ID 29 result in the ambient dose for the DEAP-3600 water shielding ($D_{w.\mathrm{sh}.}$) and, taking into account the exposure period of 622 h, the ambient dose rate ($R_{w.\mathrm{sh}.}$) as follows:

(3)” $$D_{w.\mathrm{sh}.}=3.9\pm 1.3\;\mathrm{mR}\;\;\mathrm{and}\;\;R_{w.\mathrm{sh}.}=6.2\pm 2.0\;\mu R/h.$$

has been updated to “In other words, the dose data from Table 1 for badge IDs from 4 to 24 and badge ID 29 result in the ambient dose for the DEAP-3600 water shielding ($D_{w.\mathrm{sh}.}$) and, taking into account the exposure period of 1342 h, the ambient dose rate ($R_{w.\mathrm{sh}.}$) as follows:

(3)” $$D_{w.\mathrm{sh}.}=3.9\pm 1.3\;\mathrm{mR}\;\;\mathrm{and}\;\;R_{w.\mathrm{sh}.}=2.8\pm 1.0\;\mu R/h.$$

In the third paragraph of Section 4, the last sentence “For that, one should use all data from Table 1, which results in Cube Hall ambient dose ($D_{\mathrm{CH}}$) and ambient dose rate ($R_{\mathrm{CH}}$) as follows:

(4)” $$D_{\mathrm{CH}}=4.6\pm 2.3\;\mathrm{mR}\;\;\mathrm{and}\;\;R_{\mathrm{CH}}=7.4\pm 3.7\;\mu R/h.$$

has been updated to “For that, one should use all data from Table 1, which results in Cube Hall ambient dose ($D_{\mathrm{CH}}$) and ambient dose rate ($R_{\mathrm{CH}}$) as follows:

(4)” $$D_{\mathrm{CH}}=4.6\pm 2.3\;\mathrm{mR}\;\;\mathrm{and}\;\;R_{\mathrm{CH}}=3.4\pm 1.7\;\mu R/h.$$

In the forth paragraph of Section 4, the phrase “exposure period was 622 h” has been updated to “exposure period was 1342 h”. In the same paragraph, the phrase “if the exposure period was extended to roughly three months” has been updated to “if the exposure period was extended to roughly six months”.

In the fifth paragraph of Section 4, the sentence “For example, for the ambient dose data presented in Table 1, 622 × 14 × 2 = 17,416 h (i.e., ≃725 days, or ≃2 years) would be required with a single detector placed sequentially at the same locations in Cube Hall”. has been updated to “For example, for the ambient dose data presented in Table 1, 1342 × 14 × 2 = 37,576 h (i.e., ≃1566 days, or ≃4 years) would be required with a single detector placed sequentially at the same locations in Cube Hall”.

## Table Correction

Table 1 is updated as appear below:

**Table 1:** The measurement results from integrating passive detectors, such as TLDs, for ultra-low-level ambient dose and dose rate at the surface of water shielding at the DEAP-3600 detector (except TLDs with badge IDs 25 and 26, which were placed next to the fire door; see Figure 2). TLD detectors that were inside badge ID 28 were placed on the deck of Cube Hall. TLD detectors located inside badge 29 were placed on top of the water shielding of the DEAP-3600 detector.

Badge ID	Rear Exposure (Milliroentgen)	Front Exposure (Milliroentgen)	Average Exposure (Milliroentgen)	Average Rate (μR/h)
4	4.4	5.2	4.8 ± 0.6	3.6 ± 0.4
11	2.8	3.0	2.9 ± 0.1	2.2 ± 0.1
12	4.1	4.1	4.1 ± 0.0	3.1 ± 0.0
13	2.2	2.0	2.1 ± 0.1	1.6 ± 0.1
16	2.0	2.3	2.1 ± 0.2	1.6 ± 0.2
17	2.5	2.7	2.6 ± 0.1	1.9 ± 0.1
19	5.5	4.9	5.2 ± 0.4	3.9 ± 0.3
20	4.8	6.1	5.4 ± 0.9	4.1 ± 0.7
23	4.1	3.7	3.9 ± 0.3	2.9 ± 0.2
24	5.4	5.4	5.4 ± 0.0	4.0 ± 0.0
25	8.7	10.4	9.6 ± 1.2	7.1 ± 0.9
26	8.9	9.2	9.1 ± 0.2	6.7 ± 0.2
28	3.4	2.9	3.1 ± 0.4	2.3 ± 0.3
29	4.2	3.9	4.0 ± 0.2	3.0 ± 0.2

The authors apologize for any inconvenience caused and state that the scientific conclusions are unaffected. The original article has been updated.
